# Supplementary material for: Translation elongation factor 1A2 is encoded by one of four closely related eef1a genes and is dispensable for survival in zebrafish
Source: Biosci Rep. 2020 Jan 31;40(1):BSR20194191. doi: 10.1042/BSR20194191 (PMC6997148; doi:10.1042/BSR20194191)
Supplement: Supplementary Figure S1 and S2 [file BSR-2019-4191_supp.pdf]

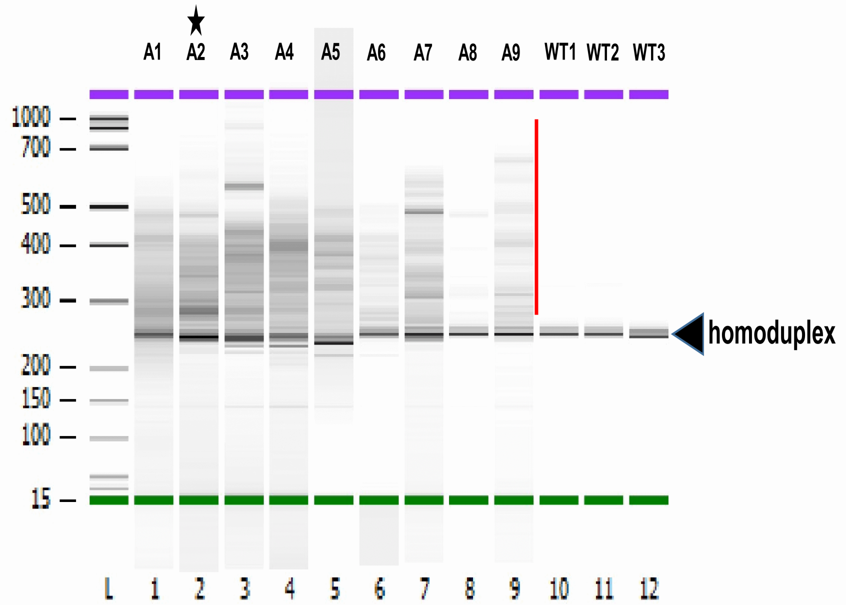

A1
A2
A3
A4
A5
A6
A7
A8
A9
WT1
WT2
WT3
1000
700
500
400
300
200
150
100
15
homoduplex
L
1
2
3
4
5
6
7
8
9
10
11
12

Supplementary figure 1 Screening of potential F0 mutants injected with gRNA by running PCR amplicons of target site on the Agilent 2100 Bioanalyser. A mismatch between wild type and mutant strands gives rise to heteroduplex ((shown by the red line)), which indicates the presence of indels in these fish which are mosaic at this stage. Black star indicates founder (F0) fish used to generate Ins4 and Del2 lines. WT1, WT2 and WT3 indicate PCR products obtained from three different uninjected wild-type fish fin-clippings.

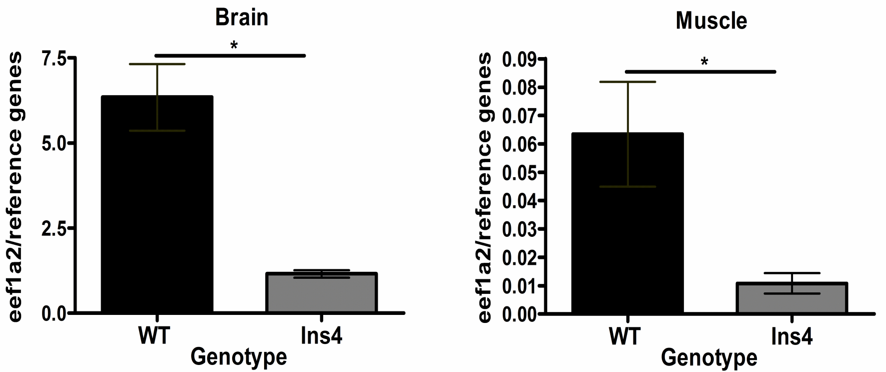
Brain
*
7.5
5.0
2.5
0.0
eef1a2/reference genes
WT
Ins4
Genotype
Muscle
*
0.09
0.08
0.07
0.06
0.05
0.04
0.03
0.02
0.01
0.00
eef1a2/reference genes
WT
Ins4
Genotype

Supplementary figure 2 Analysis of *eef1a2* transcripts in Ins4 mutants using eef1a2S. Reduced *eef1a2* transcript levels in F2 Ins4 homozygous (3 months) brain and muscle tissues was also noted using this set of primers.
